# Flexible organic synaptic device based on poly (methyl methacrylate):CdSe/CdZnS quantum-dot nanocomposites

**DOI:** 10.1038/s41598-019-46226-4

**Published:** 2019-07-05

**Authors:** Bon Min Koo, Sihyun Sung, Chaoxing Wu, Jin-Won Song, Tae Whan Kim

**Affiliations:** 1 Department of Electronics and Computer Engineering, Hanyang University, Seoul, 04763 Korea; 2 POWERLOGICS Co., Ltd., 163, Gwahaksaneop 4-ro, Oksan-myeon, Heungdeok-gu, Cheongju City, Chungcheongbuk-do Korea; 3 College of Physics and Information Engineering, Fuzhou University, Fuzhou, 350108 China

**Keywords:** Nanoscale materials, Electrical and electronic engineering

## Abstract

A synaptic device that functionally mimics a biological synapse is a promising candidate for use as an electronic element in a neuromorphic system. In this study, flexible electronic synaptic devices based on poly (methyl methacrylate) (PMMA):CdSe/CdZnS core-shell quantum-dot (QD) nanocomposites are demonstrated. The current-voltage characteristics for the synaptic devices under consecutive voltage sweeps show clockwise hysteresis, which is a critical feature of an artificial synaptic device. The effect of the CdSe/CdZnS QD concentration on the device performance is studied. The flexible electronic synaptic devices under bending show the similar and stable electrical performances. The memory retention measurements show that the e-synapse exhibits long-term potentiation and depression. The carrier transport mechanisms are analyzed, and thermionic emission and space-charge-limited-current conduction are found to be dominant.

## Introduction

Conventional digital computer architectures based on complementary metal-oxide semiconductor (CMOS) silicon technology are now facing the von Neumann bottleneck for processing data because between the memory unit and the central processing unit, a huge amount of power and time is consumed^1^. Thus, the hardware platform based on a biomimetic brain is emerging as one of the best ways to execute a neuromorphic system because such a platform offers high-speed processing and improved energy efficiency^2–8^.

The recent interest in the biomimetic brain has led to the development of a single component with synaptic features. The human brain performs learning and memory functions by utilizing enormous numbers of synapses and neurons. Although the brain is capable of handling massive amounts of information at once, it consumes very little energy. Furthermore, synapses play a significant role in the formation of processing memory with adaptable and fault-tolerant computation due to their large level of parallel connectivity. For these reasons, the development of an artificial synaptic device that function in a manner similar to a biological synapse would be a fundamental step forward in research on neuromorphic systems^9–15^.

In particular, memristive devices are promising candidates for realizing the basic function of a neuromorphic system because of their simple structure, fast operation, low fabrication cost and potential applications in achieving high-density integration^16–21^. Various kinds of materials have been reported as components for synaptic devices. Among them, polymer:quantum-dot (QD) nanocomposites have the advantage of allowing precise control of the electronic properties^22–24^. Here, we utilize a poly(methyl methacrylate) (PMMA):CdSe/CdZnS QD nanocomposite as the active layer in an electronic synaptic device because very little research has been done on such a use of this material^25–28^. In this work, flexible synaptic devices based on PMMA:CdSe/CdZnS core-shell QD nanocomposites were investigated. The current-voltage (I-V) characteristics of the synaptic devices exhibited pinched hysteresis under consecutive positive and negative voltage sweeps, and the electrical characteristics were found to depend significantly on the CdSe/CdZnS concentration. In addition, the carrier transport mechanisms at play in the electronic synaptic devices were studied.

## Experimental Details

A mixed solution with CdSe/CdZnS QDs (Powerlogics, Nanodot-HE-530) and a PMMA (Sigma Aldrich, 200336-50 G) suspension in toluene were prepared to fabricate the PMMA:CdSe/CdZnS QD nanocomposite layer. The purpose of using PMMA for the active layer of the electronic synaptic device is to reduce the operation current, which is good for reducing power consumption^16,29^. Mixed PMMA:CdSe/CdZnS QD solutions with different weigh ratios of CdSe/CdZnS QDs to PMMA were prepared by using ultrasonication for 4 h to investigate the effect of the QD concentration on the device performance. The weight of the PMMA and the amount of toluene used in this work for all devices were 100 mg and 20 ml, respectively. The same weight of the PMMA suspension and the same amount of toluene, but different amounts of CdSe/CdZnS QDs, were used to fabricate five different devices: devices 1, 2, 3, 4, and 5 for which the weights of the CdSe/CdZnS QDs in the mixed solutions were 25, 50, 75, 100, and 125 mg, respectively.

Indium-tin-oxide (ITO)-coated polyethylene glycol naphthalate (PEN) substrates were chemically cleaned by using sonication with acetone, methanol and deionized water, each for 20 min. Then, the ITO-coated PEN substrates were dried in flowing N_2_ gas. The synaptic devices were fabricated at room temperature in the atmosphere. Firstly, to fabricate a poly(3,4-ethylenedioxy-thiophene) polystyrene sulfonate layer (PEDOT:PSS) with uniform thickness, we spin-coated the PEDOT:PSS solution onto a cleaned ITO-coated PEN substrate at 500 rpm for 5 s, 1500 rpm for 15 s, 2500 rpm for 30 s, 1500 rpm for 15 s, and 500 rpm for 5 s in sequence for each film. To obtain PEDOT:PSS layers with different uniform thicknesses, we used the same sequence of spinning speeds, but different spinning times. After the sample had been spin-coated, it was thermally annealed at 110 °C for 30 min to remove the solvent. The PMMA:CdSe/CdZnS QD active layer was spin-coated onto the PEDOT:PSS layer at 500 rpm for 5 s, 3000 rpm for 40 s and 500 rpm 5 s. After that, the samples were thermally annealed at 130 °C for 20 min to remove the residual solvent. Finally, the top Al electrodes with diameters of 1 mm and thicknesses of 200 nm were deposited on the PMMA:CdSe/CdZnS QD layer by using thermal evaporation at a chamber pressure of 1 × 10^−6^ Torr.

The photoluminescence (PL) spectrum and the absorption spectrum for the CdSe/CdZnS QDs were measured by using a Hamamatsu C11347-11 system. All the electrical measurements were made using a digital Keithley 2400 source meter at room temperature. For measurements of the I-V characteristics, the top Al electrodes were grounded, and the bias voltage was applied to the bottom ITO electrode. Cross-sectional scanning electron microscopy (SEM) images were observed by using a Nova Nano SEM 230 system.

## Results and Discussion

**Figure 1:**
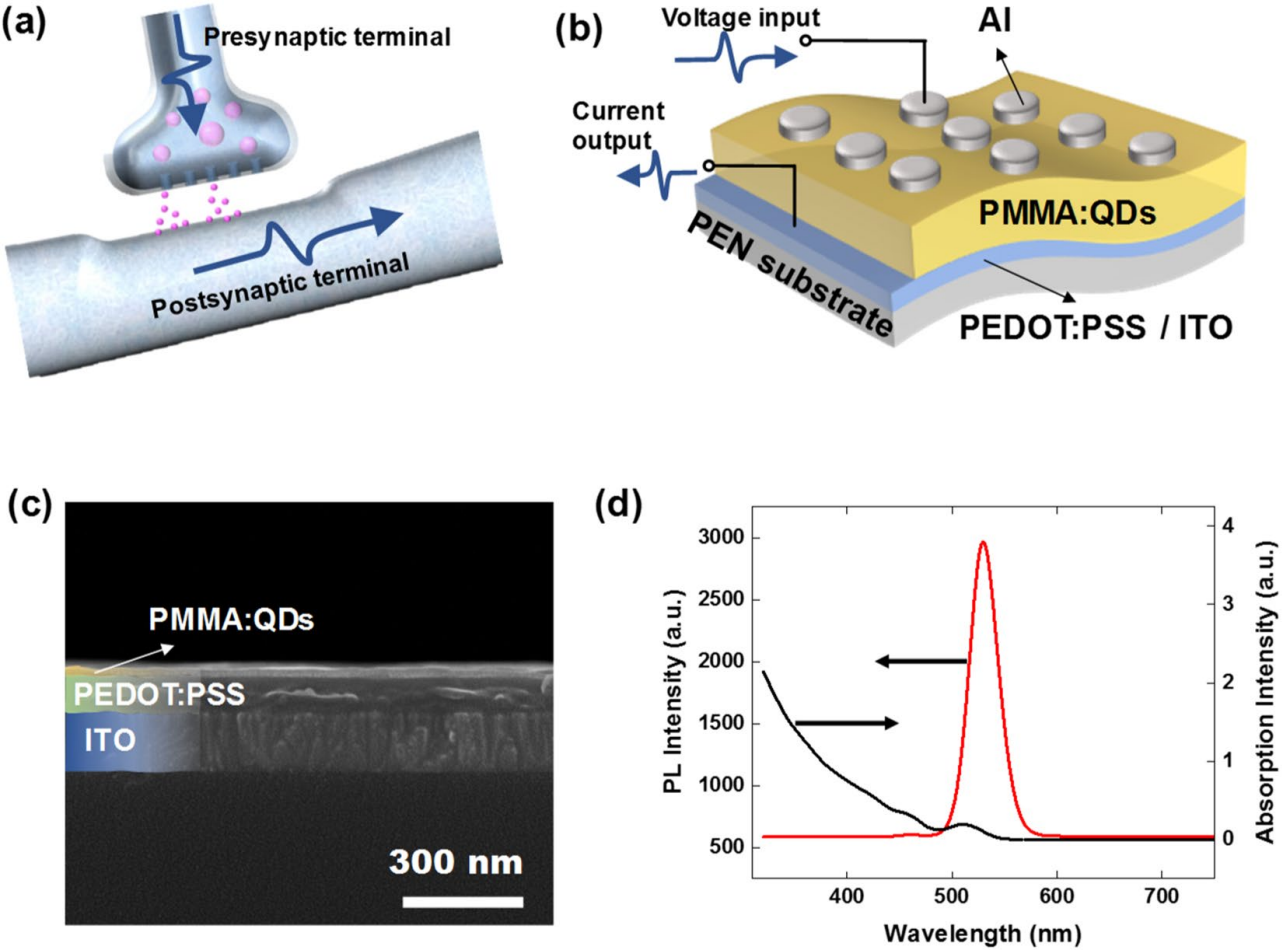
Schematics of (**a**) the neuron and the synapse and (**b**) the ITO/PEDOT:PSS/PMMA:CdSe/CdZnS QDs/Al synaptic device fabricated on a PEN substrate. (**c**) Cross-sectional SEM image of the device. (**d**) Photoluminescence (red curve) and absorption (black curve) spectra for the CdSe/CdZnS.

Figure 1(a,b) show schematics of the structure of a synapse and the structure of the synaptic device used in this work, respectively, and Fig. 1(c) shows a cross-sectional SEM image of the fabricated synaptic device. The SEM image shows that the PMMA:CdSe/CdZnS QD layer, which has a thickness of 35 nm, is uniformly deposited on the ITO bottom electrode. The PEDOT:PSS layer with a thickness of about 80 nm was used to enhance the adhesion between the active layer and the ITO electrode. Figure 1(d) presents the PL spectrum and the absorption spectrum for the CdSe/CdZnS QDs, which clearly indicate the high quality of the CdSe/CdZnS QDs used in this work.

**Figure 2:**
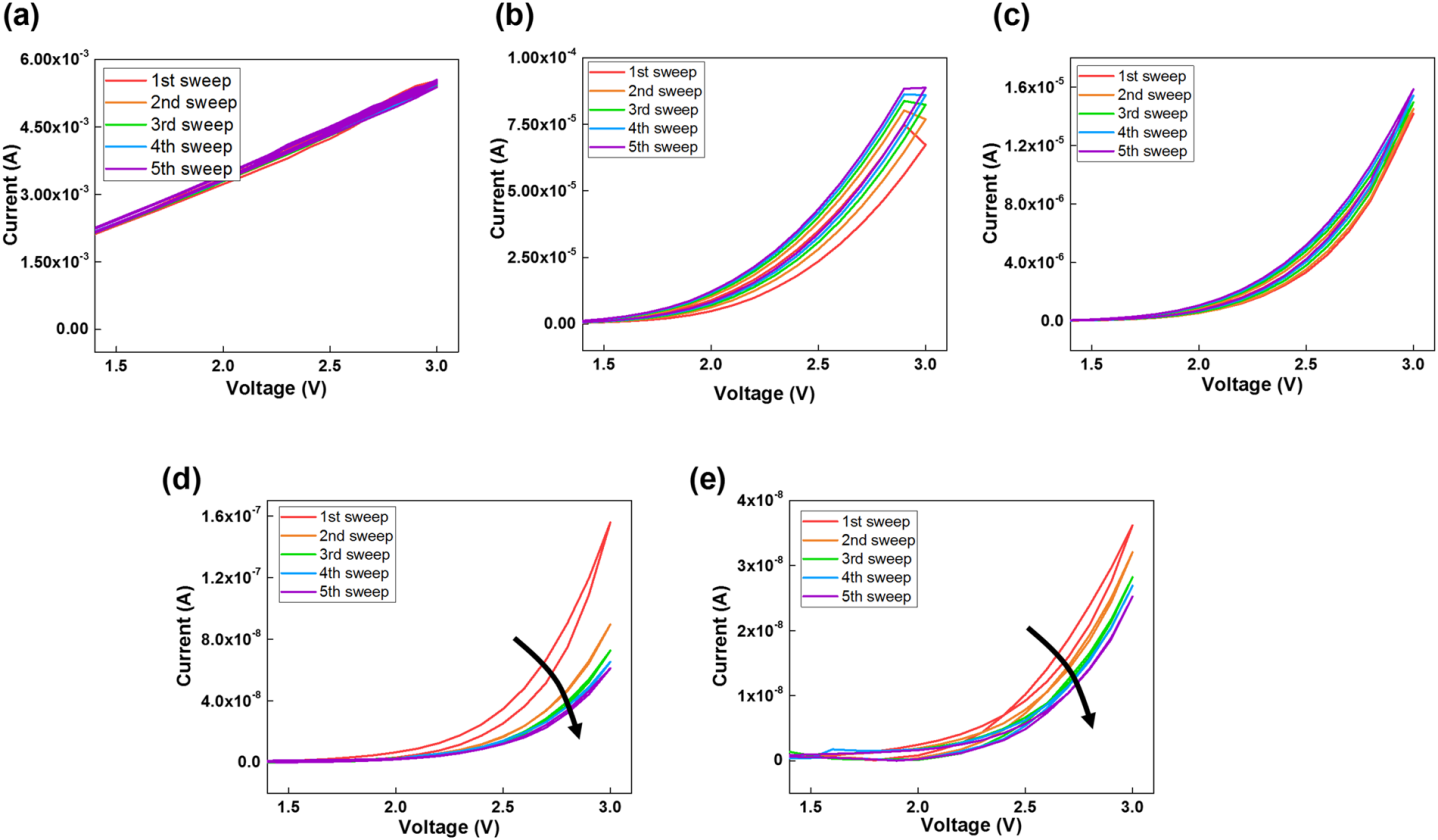
I-V characteristics after one to five voltage sweeps for the devices with various amounts of CdSe/CdZnS QDs in the PMMA matrix: (**a**) device number 1 (20 wt%), (**b**) device number 2 (33.3 wt%), (**c**) device number 3 (37.5 wt%), (**d**) device number 4 (50 wt%), and (**e**) device number 5 (62.5 wt%).

Note that synthesizing QDs with a highly uniform size-distribution is difficult. Thus, we only focus on the effects of the number of CdSe/CdZnS QDs in the nanocomposite on the device performance. The effects of the amount of CdSe/CdZnS QDs on the device performance were studied by measuring the I-V characteristics of the synaptic devices for various amounts of PMMA:CdSe/CdZnS QDs, and the results are shown in Fig. 2(a–e) for devices 1, 2, 3, 4, and 5 with 20, 33.3, 42.9, 50, and 55.6 wt% of CdSe/CdZnS QDs in the nanocomposite layers, respectively. For the current measurements, five consecutive positive voltage sweeps were applied to the synaptic devices. As shown in Fig. 2, the amount of CdSe/CdZnS QDs in the PMMA matrix clearly has an effect on the electrical properties of the synaptic devices. Device 1 under dual voltage (0–3–0 V) sweeps showed a relatively large current in comparison with other devices, and the shape of its I-V appears to be linear, as shown in Fig. 2(a). However, device 1 cannot be used as synaptic device because the difference in current between five consecutive applied voltage sweeps is inadequate. For devices 2 and 3 shown in Fig. 2(b,c), respectively, the current consistently decreases with increasing number of voltage sweeps. However, the current differences for devices 2 and 3 are smaller than that of device 4 shown in Fig. 2(d). For device 4, the maximum current clearly decreases with increasing number of dual voltage sweeps, with the maximum currents at 3 V for five consecutive voltage sweeps being 1.56 × 10^−7^, 8.95 × 10^−8^, 7.25 × 10^−8^, 6.52 × 10^−8^, and 6.10 × 10^−8^ A, respectively. The dual voltage sweeps in Fig. 2(d) show clockwise hysteresis, which is a critical feature of an artificial synaptic device. In Fig. 2(e), the dual voltage sweeps for device 5 show that the maximum current at 3 V is decreased and the pinched hysteresis is unstable. Especially, as shown in Fig. 2(a–e), the highest currents in the synaptic devices with different amounts of QDs steadily decrease with increasing amount of CdSe/CdZnS QDs in the active hybrid layer. Furthermore, the I-V data for the dual voltage sweeps with synaptic device 4 show excellent pinched hysteresis behavior similar to that of a biological synapse. Therefore, in the following discussion, we use the data for device 4 for a detailed analysis of the device characteristics.

**Figure 3:**
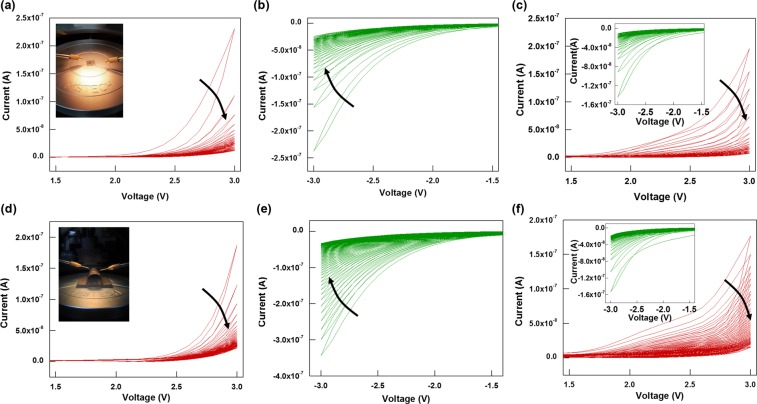
(**a**) I-V curves for the first stimulation of the flat device (inset) under consecutive (**a**) positive and (**b**) negative voltages. (**c**) I-V curves for the second stimulation of the flat device under consecutive positive voltages. The inset presents the I-V curves for the second stimulation under consecutive negative voltages. I-V curves for the first stimulation of the bent device (inset) under consecutive (**d**) positive and (**e**) negative voltages. (**f**) I-V curves for the second stimulation of the bent device under consecutive positive voltages. The inset presents the I-V curves for the second stimulation under consecutive negative voltages. In all the figures, the arrows are in the direction of increasing number of voltage sweeps.

A more specific operation of the ITO/PEDOT:PSS/PMMA:CdSe/CdZnS QDs/Al synaptic device is shown in Fig. 3. Figure 3(a–c) show the I-V characteristics for the flat (before bending) synaptic device under consecutive positive and negative voltage sweeps. Figure 3(a) shows the I-V curves when consecutive positive voltage sweeps are applied to the synaptic device. The inset is a picture of the flat device. The dual voltage sweeps of the synaptic device show clockwise pinched hysteresis behavior. The electrical characteristics of the device show a low-resistance state in the forward sweep and a relatively high-resistance state in the reverse sweep. As a result, during several dual voltage sweeps, the conductivity of the device consistently decreases. While consecutive positive voltage (0–3–0 V) sweeps are being applied, the current at 3 V decreases from 2.31 × 10^−7^ to 1.20 × 10^−8^ A. This behavior is similar to the depression behavior in a biologic synapse. After the positive voltage sweeps have been applied, negative consecutive voltage (0–3–0 V) sweeps are applied to the synaptic device, and the results are shown in Fig. 3(b). The dual voltage sweeps of the synaptic device also show a clockwise pinched hysteresis behavior. As with Fig. 3(a), the conductivity of the synaptic device continually decreases with increasing number of negative voltage sweeps. The current in the synaptic device under consecutive negative voltage sweeps at −3 V decreases from 2.38 × 10^−7^ to 2.44 × 10^−8^ A. After the first positive-negative voltage stimulations, another series of positive voltage sweeps was applied to the device. Figure 3(c) shows the I-V curves for the synaptic device under positive voltages after the negative voltage stimulation in Fig. 3(b). The device’s conductance under the positive voltage sweep (Fig. 3(a)) can be seen to be decreased; however, after the ensuing negative voltage stimulation (Fig. 3(b)), the original high conductivity can be seen to have been recovered. Similarly, in Fig. 3(c), the current under the consecutive positive voltage sweeps at 3 V decreases from 1.95 × 10^−7^ to 7.12 × 10^−9^ A. The inset presents the I-V curves under consecutive negative voltages sweeps, and the current at −3 V decreases from 1.44 × 10^−7^ to 1.10 × 10^−8^ A. When the I-V curves for the first positive-negative voltage stimulation are compared, the maximum currents are found to be similar, indicating that the synaptic device is rewritable. Figure 3(d–f) present data showing the stability of the synaptic device under bending. This flexible synaptic device is clearly stable under bending because the pinched hysteresis behaviors and the electrical characteristics of the bent device are similar to those for the flat device.

**Figure 4:**
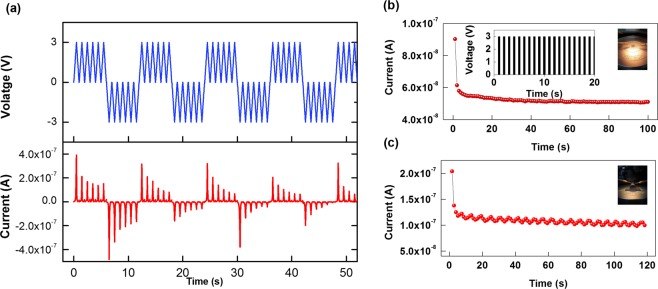
(**a**) Current and voltage as functions of time to establish the endurance characteristics under consecutive voltages applied to the device. I-t curves for the device under pulse voltage for the device in a (**b**) flat state and (**c**) the bend state. The inset in (**b**) presents the pulse voltage applied to the device.

Figure 4(a) shows a series of voltage stimulations with five consecutive positive voltage sweeps followed by five consecutive negative voltage sweeps being applied to the synaptic device. Set and Reset conditions can be seen in V-t and the I-t data. Thus, one can conclude that the synaptic device is rewritable with repeated switches between low-resistance and high-resistance states. Figure 4(b,c) present the I-t curves when a 100 cycles of pulse voltage (3 V, 100 ms) are applied to the flat and the bent device, respectively. The inset of Fig. 4(b) presents the pulse voltage applied to the electronic synaptic device. As shown in Fig. 4(b,c), the current in the flat device under pulse voltage decreases from 9.03 × 10^−8^ to 5.11 × 10^−8^ A and that in the bent device decreases from 2.04 × 10^−7^ to 9.98 × 10^−8^ A. Note that the final current in the bent state is a little higher than that in the flat state. A possible reason is that under bending, the PMMA:CdSe/CdZnS QD layer is stretched. As a result, the thickness of the active layer is reduced, and the distance between neighboring QDs in the vertical direction is reduced, leading to an increase in the current.

**Figure 5:**
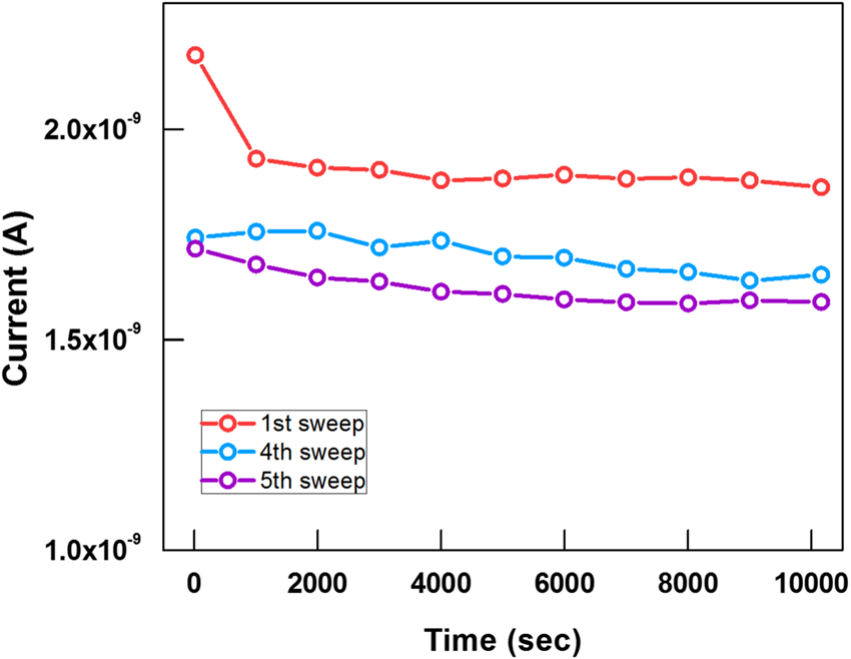
Retention characteristics (I-t) of the device after the first, fourth and fifth voltage sweeps at a reading voltage of 0.3 V.

Figure 5 shows the retention characteristics of the ITO/PEDOT:PSS/PMMA:CdSe/CdZnS QDs/Al synaptic device measured at room temperature and a reading voltage of 0.3 V after one, four, and five consecutive positive voltage sweeps have been applied to the device. As shown in the graphs, the currents at different conductivity states are maintained for up to 10^4^ s, indicating that the ITO/PEDOT:PSS/PMMA:CdSe/CdZnS QDs/Al synaptic device has a high stability. These results indicate that the depression of our device can be attributed to a long-term depression behavior. Note that the current drops significantly in 1000 s for the first sweep. A possible reason is that the electrons are more easily captured by the QDs because the QDs have previously only captured a small number of electrons. As a result, the current drops significantly in the beginning. The result is similar to the I-V cure shown in Fig. 2(d), in which the current decreases significantly in the beginning. However, for other cases after the fourth and the fifth positive voltage sweeps have been applied to the device, the capture of electrons by the QDs is more difficult because the QDs have already captured a significant number of electrons.

**Figure 6:**
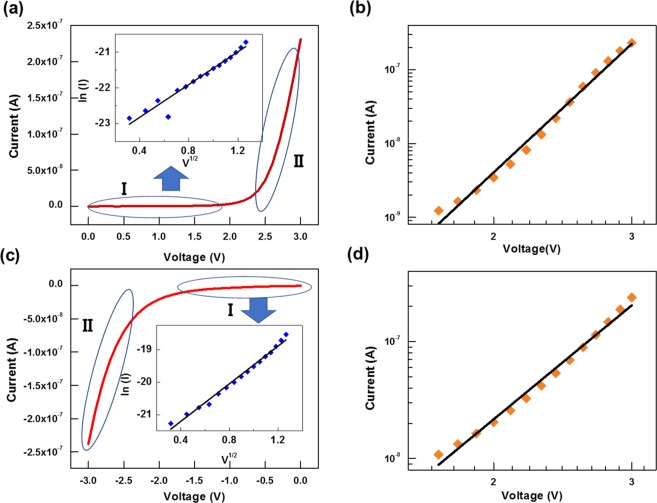
(**a**) I-V fitting to a ln (I) versus V^0.5^ curve for the device under a positive voltage from 0 to 3 V. The inset presents the I-V fitting to the ln (I) versus V^0.5^ curve for the device under a positive voltage from 0 to 1.6 V (region I in (**a**)). (**b**) I-V fitting to a ln (I) versus ln (V) curve under a positive voltage from 1.7 to 3 V (region II in (**a**)). (**c**) I-V fitting to a ln (I) versus V^0.5^ curve for the device under a negative voltage from 0 to −3 V. The inset presents the I-V fitting to the ln (I) versus V^0.5^ curve for the device under a negative voltage between 0 to −1.6 V (region I in (**c**)). (**d**) I-V fitting to a ln (I) versus ln (V) curve under a negative voltage from −1.7 to −3 V (region II in (**c**)).

The I-V characteristics of the synaptic device were studied further to understand the carrier transport mechanisms at work in the device, and the results are presented in Fig. 6. The results obtained by fitting the first sweep of the I-V curves are presented in Fig. 3(a,b). We used the field-assisted thermionic emission model (Eq. 1) and the space-charged-limited-current (SCLC, Eq. 2) model to fit the *I-V* curves^30,31^:

1 $${\rm{I}}\propto {\rm{A}}{T}^{2}\exp [-\frac{q\phi }{kT}+q{(\frac{{q}^{3}V}{4\pi \varepsilon })}^{1/2}],$$

2 $${\rm{I}}\propto {V}^{\alpha },$$

where I, V, A, T, ε, φ, k, and q represent the current, applied voltage, Richardson’s constant, absolute temperature, dielectric permittivity, barrier height, Boltzmann’s constant, and electronic charge, respectively.

Figure 6(a) shows the I-V curve when a positive voltage (0–3 V) is applied to the electronic synaptic device. The inset shows the fitted I-V graph under a positive consecutive voltage sweep, and a linear relationship between ln (I) and V^1/2^ is seen at voltages from 0 to about 1.6, indicating that the carrier transport in the synaptic device is dominated by thermionic emission in region I of the graph in Fig. 6(a). On the other hand, for region II of the graph in Fig. 6(a), as shown in Fig. 6(b), the slope of ln (I) versus ln (V) positive voltage curve for voltages from 1.7 to 3 V is 9.88, indicating that the carrier transport in the synaptic device is dominated by SCLC conduction, which results from the electron capture by QDs. Figure 6(c,d) show the fitted graphs under negative consecutive voltage (0–3 V) for the first sweeps. The linear relationship between ln (I) and V^1/2^ at voltages from 0 to −1.6 V (region I), as seen in the inset of Fig. 6(c), and the linear relationship between ln (I) and ln (V) at voltages from −1.7 to −3.0 V (region II), as seen in Fig. 6(d), indicate that the mechanisms are the same as those in Fig. 6(a,b).

## Conclusion

Flexible electronic synaptic devices based on PMMA:CdSe/CdZnS QD nanocomposites were fabricated. We observed that the current differences in the devices depended significantly on the CdSe/CdZnS QDs concentration in the nanocomposites. The I-V characteristics of the synaptic devices showed clockwise pinched hysteresis behavior similar to a biological synapse. Furthermore, the synaptic devices exhibited excellent bending stability because the electrical characteristics after bending were similar to those for the devices in the flat state. The carrier transport mechanisms in the synaptic devices were dominated by thermionic emission and SCLC conduction.
